# Infiltration of the Hoffa's fat pad with stromal vascular fraction in patients with osteoarthritis of the knee -Results after one year of follow-up-

**DOI:** 10.1016/j.bonr.2022.101168

**Published:** 2022-01-22

**Authors:** Klaus Werner Labarre, Gerald Zimmermann

**Affiliations:** Theresienkrankenhaus St. Hedwigklink GmbH, Germany

**Keywords:** MSCs, Mesenchyaml stem cell, HIF, Hypoxia-inducing factor, ADSC, Adipose-derived stem cell, IFP, Infrapatellar fat pad, BMSC, Bone marrow-derived stem cells, SVF, Stromal vascular fraction, VAS, Visual Analog Scale, KOOS, Knee Knee Injury and Osteoarthritis Outcome Score, VR 12, Veterans RAND 12, MCS-12, Mental component score, PCS-12, Physical component score, DAPI, 4′,6-Diamidine-2-phenylindole, ISCT, International Society for Cellular Therapy, CFU-F, Colony-forming units - fibroblasts, Knee, Osteoarthritis, MSC, SVF, Intra-articular injection, Cell therapy

## Abstract

**Objectives:**

Cell therapy using multipotential stromal cells (MSCs) is being used in a variety of clinical settings to induce tissue regeneration. Promising results have also been achieved in the therapy of osteoarthritis. MSCs have been demonstrated to be safe (Borakati et al., 2018). They can be used in a one step procedure as minimally manipulated mesenchymal stem cells or after in vitro expansion. The in vitro step allows for the selection of a more homogeneous cell population, meeting the standard criteria for MSC identification (Lv et al., 2014). In vitro expansion of MSCs is cost intensive, time consuming and furthermore associated with gradual accumulation of senescent cells (Wagner et al., 2008), telomere erosion (Baxter et al., 2004), and changing phenotypes (Jones et al., 2010; Halfon et al., 2011). These disadvantages could be surpassed by the use of “minimally manipulated mesenchymal stem cells” from bone marrow or adipose tissue (Di Matteo et al., 2019) such as the adipogenic stromal-vascular fraction (SVF).

The study investigates whether infiltration of the Hoffa fat pad with autologous SVF is an effective and safe treatment option for patients with gonarthrosis. Furthermore, the number and vitality of the injected cells as well as the clinical efficacy will be evaluated.

**Materials and methods:**

We conduct a prospective study. Patients with osteoarthritis of the knee receive infiltration of SVF into the Hoffa fat pad. The number and vitality of the cells are measured with a cell counter. The clinical outcome is checked using VAS, KOOS and SF12 questionnaires with a follow-up period of 1 year.

**Results:**

A total of 33 patients and 36 knees were included in this Study. An average of 45 million cells were injected with a standard deviation of 2,5 million Cells. After 6 months a significant improvement of the VAS and the respective subscales of the KOOS could be observed compared to the baseline. After one year of follow-up, a significant improvement in all KOOS subscales compared to baseline was still observed. A significant correlation between reduced knee pain on the VAS and the number of injected cells could be observed as well. Thus, patients injected with a higher number of cells seem to have a better outcome. The average viability of the cells was 64,4% with a standard deviation of 15,9%. A correlation between higher cell viability and better outcome on the QOL subscale of the KOOS was observed. There were no major complications or side effects.

**Discussion:**

These initial results indicate that treatment with SVF is a safe therapeutic option that has the potential to relieve joint pain and significantly improved function. The cell number and vitality of the injected cells appear to be important factors influencing the success of the therapy.

## 1 Introduction

Gonarthrosis shows a growing prevalence. It is the fourth most common cause of disability worldwide and is therefore of enormous socioeconomic relevance (Brooks, 2002; Fransen et al., 2011).

With a constantly aging population, the prevalence of gonarthrosis is expected to rise continuously (Issa and Sharma, 2006). Treatment with knee arthroplasty in the final stage of the disease is frequently the last option.

Established conservative therapies for the treatment of gonarthrosis are limited to symptom control rather than causative treatment [26]. These therapies can usually provide only moderate symptom relief for patients, but are associated with undesirable side effects (Abraham et al., 2007; Bagga et al., 2006; Hawker et al., 2011; McAlindon et al., 2014).

Pathophysiological studies have shown that the catabolic, pro-inflammatory and anabolic processes taking place in the affected joint play a very important role in the course of the disease (Goldring, 2000). The oxygen radicals released in response to mechanical stress cause chondrocyte senescence (Jeon et al., 2018). This leads to a pro-catabolic state which promotes the progression of arthrosis (Ankrum et al., 2014). It is assumed that Mesenchymal stem cells (MSCs) are able to counteract this process. Furthermore multiple recent studies describe that the infrapatellar fat pad (IFP) and synovium play a key role in the onset and progression of joint disease by organizing immunological and inflammatory processes and thus make the IFP a potential therapeutic target for cell-based osteoarthritis therapy. The IFP serves as the origin of pro-inflammatory and articular cartilage degrading mediators. It is also origin of the substance P which is secreted by sensory nerve fibers and is associated with pain transmission and modulation of local inflammatory/immune and fibrotic response. Substance P could be neutralized by the enzymatic activity of surface neutral endopeptidase CD10 which is expressed in multiple cell types including MSCs (Lv et al., 2014; Ong et al., 2014; Maguer-Satta et al., 2011; Xie et al., 2011). Beneath stromal cells the IFP also contains immune related cells including Monocytes/Macrophages. These Macrophages can be polarized into the M1 (classical pro-inflammatory) and M2 (alternative anti-inflammatory) polarization phenotypes (Ginhoux and Jung, 2014; Greif et al., 2020).

Several studies indicate that the polarization of pro-inflammatory (M1) macrophages into theM2 anti-inflammatory phenotype is a relevant part of the immunmodulating features of MSCs (Abumaree et al., 2013; Prockop, 2013; Ylöstalo et al., 2012).

MSCs are multipotent stem cells derived from the mesoderm, which have the ability to differentiate into osteoblasts, chondroblasts, chondrocytes, myocytes and adipocytes (Caplan, 2007). Although the mechanism of cartilage regeneration by MSCs has not yet been clarified, it is assumed that two harmonizing mechanisms play an important role in this process. Direct adherence and incorporation of MSCs into the host tissue for growth and differentiation and/or trophic effects resulting from the secretome of MSCs (Zwolanek et al., 2017).

After MSC have been injected, they can migrate to the corresponding target tissue through interaction with various chemokine receptors. This is called the “homing” effect (Khaldoyanidi, 2008; Sohni and Verfaillie, 2013). Animal studies showed that intra-articularly injected MSCs migrate to the site of the cartilage defect and engraft to the synovial membrane in order to induce regeneration (Park et al., 2017; Mizuno et al., 2008; Kouroupis et al., 2019). Further studies showed that although the binding of MSCs to the cartilage defect is necessary, the attached MSCs coordinate the regeneration process rather than transforming themselves into new chondrocytes (Zwolanek et al., 2017; de Windt et al., 2017).

The bioactive factors secreted by MSCs can be categorized into three classes. Growth factors Cytokines and extracellular vesicles (Arslan et al., 2013; Lai et al., 2010; Lai et al., 2013). MSCs produce anti-inflammatory cytokines (Puetzer et al., 2010). Among these secreted cytokines, hypoxia-inducing factor (HIF) is thought to have the ability to promote chondrogenesis (Cantinieaux et al., 2013; Amann et al., 2017) and the insulin-like growth factor-1 (IGF-1) to promote MSC proliferation and differentiation (Lee et al., 2011). Furthermore MSCs prevent the death of chondrocytes by the expression of anti-apoptotic proteins and by the stimulation of inhibitor proteins of apoptosis (Puetzer et al., 2010). They also inhibit the production of proapoptotic factors and stimulate the production of antiapoptotic factors (Shang et al., 2014). Secreted extracellular vesicles may contribute to cartilage regeneration through paracrine-like actions (Rani et al., 2015; Tan et al., 2021).

The currently available data support the notion that a variety of growth factors and cytokines produced by MSCs work together to promote cartilage tissue regeneration.

In preclinical animal studies, pain relief and improved function have already been observed after intra-articular injection of MSCs into arthritic joints (Kouroupis et al., 2019; Jo et al., 2014; Murphy et al., 2003; Lee et al., 2007). Clinical studies have shown comparable results in the treatment of knee joint arthrosis with MSCs (Jo et al., 2014; Lee et al., 2007). Phase 1 and 2 clinical trials in which patients were injected with different doses of in vitro expanded MSCs demonstrated a reduction in cartilage defects both radiologically and in arthroscopic follow-up (Freitag et al., 2019; Lu et al., 2019).

MSC exist in various tissues of the body and are usually taken from bone marrow or fatty tissue.

Accordingly, adipose-derived stem cells (ADSCs) are distinguished from bone marrow-derived stem cells (BMSCs), which have pathophysiological comparable properties.

Initially in 2006 the International Society for Cellular Therapy (ISCT) proposed three minimal criteria for defining MSCs. They should be plastic adherent, expressing a panel of key markers including CD105, CD73 and CD90 while being negative for CD45 and CD34. Furthermore they should be able to differentiate into osteoblasts, adipocytes and chondroblasts (Dominici et al., 2006).

In contrast to cultured MSCs fewer information is available about the tissue precursor cells that give rise to plastic adherent cells. In an attempt to clarify the terminology, the ISCT proposed in a position paper to refer to cultured MSCs as “multipotent mesenchymal stromal cells”. The term “mesenchymal stem cells” should be reserved for cells from primary tissues that can give rise to “colony-forming units - fibroblasts (CFU-F)” in vitro and a tissue repopulation with the capacity for multilayer differentiation in vivo (Horwitz et al., 2005). Several studies report strong phenotypical evidence that CD45^−/low^ CD271^bright^ (CD271) cell population represents the candidate BM-MSC population in vivo (Jones et al., 2002; Jones et al., 2006; Bühring et al., 2007; Lv et al., 2014; Tormin et al., 2011). “Culture-expanded MSCs” generated from FACS- purified CD271 cells have a molecular profile identical to that of culture-expanded MSCs generated from standard plastic adherence (Churchman et al., 2012).

Cuthebert et al. found a close linear relationship between the manually counted CFU-F colonies after culturing BM for 14 days and the number of CD271^bright^ Cells per ml of bone marrow aspirate (Cuthbert et al., 2012).

The revised statement published by The International Federation for Adipose Therapeutics and Science (IFATS) and the ISCT in 2013 proposed that the freshly isolated uncultured adipose stromal cell population, containing native ADSCs, is characterized as CD45^−^, CD235a^−^, CD31^−^, and CD34^+^ cells (Bourin et al., 2013). Compared to BM-MSCs which constitute a rare population 0.001%–0.002% of the total stromal cell population in bone marrow the amount of ASCs in freshly isolated SVF is much higher sometimes up to 30% (Caplan, 2007; Lataillade et al., 2017; Mazini et al., 2019). However, ADSCs are easier to obtain and have a lower morbidity at collection (Pers et al., 2016).

MSC can be obtained by cultivation and in vitro expansion. This process is very time consuming and cost intensive, whereas the stromal-vascular fraction (SVF) can be obtained by mechanical or enzymatic processing within 1 h after the collection of the adipose tissue. MSC products produced by culture expansion often have a high number of nucleated cells with high proportion of MSCs compared to SVF. However, studies have shown that cells manipulated in this way may lose their homing effect and are associated with gradual accumulation of senescent cells, telomere erosion, and changing phenotypes. (Khaldoyanidi, 2008; Sohni and Verfaillie, 2013; Baxter et al., 2004; Halfon et al., 2011; Jones et al., 2010; Wagner et al., 2008). This may adversely affect their therapeutic efficacy. Furthermore, culture expanded cells are classified as ATMP (Advanced Therapy Medicinal Product), which are subject to strict regulations in Europe (EMA/CAT/852602/2018). The stromal-vascular fraction (SVF) can be isolated and applied by minimal manipulation which means that it is not initially classified as an ATMP. Approval by the regional councils is nevertheless required (Lopa et al., 2019).

The SVF contains regenerative cells such as ADSCs, macrophages, blood cells, pericytes, fibroblasts and vascular cells such as endothelial cells, smooth muscle cells and the respective progenitor cells (Bourin et al., 2013). However, compared to in vitro expanded ADSCs, the number of MSCs detectable in SVF is less constant and usually lower, although this does not necessarily imply a lower clinical efficacy.

The safety and efficacy of SVF cells have been evaluated in various clinical settings including cardiology, urology, plastic and reconstructive surgery, and orthopedics (Alatab et al., 2019; Lasso et al., 2018; Borakati et al., 2018; Di Matteo et al., 2019). Initial studies have already shown promising results after intra-articular injection for the treatment of gonarthrosis. The injection of MSCs is considered a safe procedure (Tsubosaka et al., 2020; Labarre and Zimmermann, 2021).

In a prospective case series, we report first results of an injection with SVF in gonarthrosis with regard to pain reduction and quality of life. Furthermore, the number and viability of the actually injected MSCs were examined. Due to the current legal situation, intra-articular injection of SVF in Europe is currently only permitted in the Hoffa's fat pad (rev.1 EC, 2015). However, it can be assumed that the cells will be able to reach the target area due to the homing effect.

## 2 Materials and methods

### 2.1 Study design

This is a prospective single-center study to evaluate the efficacy of SVF therapy in patients with knee osteoarthritis. All described human studies have been conducted with the approval of the responsible Ethics Committee, in accordance with national law, and in accordance with the Declaration of Helsinki of 1975 (in the current, revised version). The therapy has been approved by the responsible regional council. A declaration of consent has been obtained from all patients involved.

### 2.2 Patients

Patients receiving therapy with “SVF” were invited to participate in the study. Included were all male and female patients from the age of 18 years with a Kellgren-Lawrence score up to 4. Exclusion criteria were patients with malignant tumors, sepsis and patients with skin lesions at the site of collection or injection.

All patients were registered in the SOS (Surgical Outcome System) database of Arthrex GmbH (Naples, FL, USA) after prior consent. 4 patients had to be excluded due to incompliance to the follow up schedule.

### 2.3 Liposuction, preparation of fatty tissue and preparation of SVF production

**Fig. 1:**
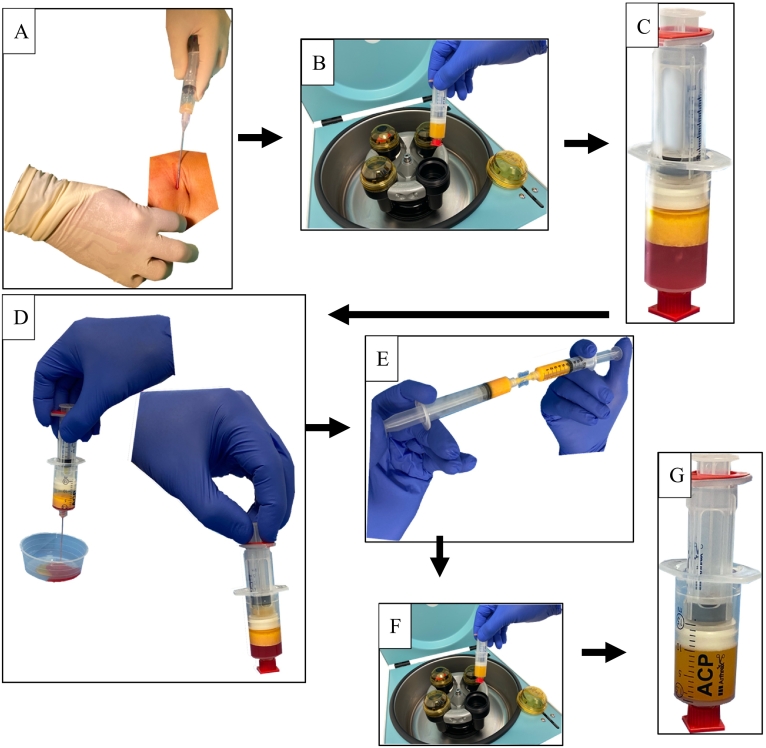
(A) Harvesting of the abdominal fat tissue, (B) centrifugation of the fat tissue at 2500 rpm for 4 min, (C) lipoaspirate after the first centrifugation with aqueous fraction at the bottom, the fat graft in the middle and a layer of oil above it. (D) The aqueous fraction is removed and the oil is transferred into the small syringe and removed as well. (E) The fat graft was transferred into two 10 ml Luer-Lock syringes and then transferred at least 30 times from one syringe to the other for homogenization using a 1.4 mm connector. (F) Second centrifugation at 2500 rpm for 4 min. (G) After the second centrifugation an SVF Pallet of approximately 1 ml can be observed at the bottom of the double syringe with a layer of oil form the destroyed adipocytes above it.

The injection of SVF was performed on an outpatient basis under sterile conditions. In order to obtain the lipoaspirate necessary for the production of SVF, the patient was injected with tumescent anesthesia and lipoaspirate was removed from the abdominal fat tissue Fig. 1(A). Under sterile conditions, an infiltration of 150 ml tumescent solution consisting of 50 ml prilocaine, 1 ml epinephrine, 6 ml sodium hydrogen carbonate and 1000 ml sodium chloride 0.9% was performed.

Using the Arthrex ACA Kit® (Arthrex GmbH, Naples, FL, USA), 30 ml of lipoaspirate was collected from the lower abdomen in two Arthrex ACP® double syringes. A Carraway Harvester ® (Tulip Medical Products (San Diego, CA, USA))^1^ was connected to the syringes The Arthrex ACP® double syringe consists of a large and a small syringe which is located in the plunger of the large syringe. The small syringe can be used to remove a liquid fraction that is above a solution after centrifugation without contaminating the remaining or the removed product. In the next step, the lipoaspirate, divided into 15 ml portions per double syringe, was centrifuged at 2500 rpm in a centrifuge (Rotofix 32A® (Andreas Hettich GmbH & Co. KG, Tuttlingen, Germany) from Hettich Centrifuges) for 4 min at room temperature Fig. 1(B).

The lipoaspirate is divided into oil, fat graft and an aqueous fraction Fig. 1(C). The oil was transferred to the small syringe and discarded. The aqueous fraction was removed Fig. 1(D).

The fat graft was transferred into two 10 ml Luer-Lock syringes and then transferred at least 30 times from one syringe to the other for homogenization using a 1.4 mm connector Fig. 1(E).

Approximately 20 ml of fat graft per collection could be isolated and transferred 30 times from one syringe to the other for further processing. The fat graft was then centrifuged again at 2500 rpm for 4 min. Afterwards, a pellet of approx. 1 ml in size could be seen at the bottom of the syringe, which contained the SVF. Above it was a layer of oil from the destroyed adipocytes, which was aspirated by the small syringe Fig. 1(G).

For better application of the SVF, it was diluted with 5 ml NaCl 0.9% solution. Before injection, a sample of SVF was taken to determine the number of nucleated cells and the proportion of vital cells. The stromal-vascular fraction was injected into Hoffa's fatty body under sonographic control. The whole procedure from fat extraction to injection took about 1 h.

Patients could be mobilized and discharged immediately after injection of the SVF. No further physiotherapeutic treatments or interventions were performed in the postoperative period.

### 2.4 Cell count

The cell count was determined by using a NucleoCounter NC-200® cell counter (ChemoMetec A/S, Allerod, DK, Denmark). An untreated sample was filled into a Via 1 cassette, which stained nucleoli of dead cells with 4′,6-Diamidine-2-phenylindole (DAPI). The number of nucleoli of dead cells was determined by the cell counter. A second portion of the sample was treated with Reagent A100 and Reagent B, which leads to lysis of the cell membranes. In the sample pretreated in this way all cells were stained with DAPI and the total number of cells was determined by the cell counter. From the difference the percentage of vital cells was calculated, which is automatically indicated one percent by the cell counter. The injected volume was recorded for each patient. Thus, the number of actually injected cells and the percentage of vital cells could be calculated. The time between fat harvesting and Cell analysis varied between 1 and 4 h while the samples were stored at room temperature.

### 2.5 Clinical outcome

Clinical progress was monitored using the Visual Analog Scale (VAS), Knee Injury and Osteoarthritis Outcome Score (KOOS) and the SF 12. To determine the baseline values, all questionnaires were completed by the patient on the day of treatment. Further questionnaires were automatically sent to the patients by the SOS via e-mail at intervals of 2 weeks, 6 weeks, 3 months 6 months and 1 Year (Table 1).

**Table 1:** Intervals for outome measurements.

Follow up schedule	Follow up time points						
Outcomescore	Pre-treatment	1 week	2 weeks	6 weeks	3 month	6 month	1 Year
VAS	X	X	X	X	X	X	X
KOOS	X				X	X	X
VR 12 Physical	X					X	X
VR 12 mental	X					X	X

#### 2.5.1 Visual analog scale (VAS)

VAS is an instrument for measuring subjective pain intensity. Here, patients enter their pain on a vertical line. The ends of this line represent extreme values. Left: “no pain” and right: “extreme pain”. The given values are quantified with points from 1 to 10 (Thong et al., 2018).

#### 2.5.2 “Knee injury and osteoarthritis score” (KOOS)

The Knee injury and osteoarthritis score (KOOS) is a tool to evaluate clinical limitations in patients with knee joint arthrosis, which has been validated in numerous studies.

The KOOS consists of 5 subscales:

• Pain (pain)
• Symptoms
• activities of daily living (ADL)
• Function in sport and recreation Sport/Rec
• Quality of life related to the affected knee (knee related Quality of life - QOL)

The patients have to answer the 42 questions at fixed times. The questions are assigned a point value, whereby a total point value of 0–100 is to be achieved. 100 points mean almost no restrictions and 0 points mean maximum possible restrictions (Collins et al., 2016).

#### 2.5.3 VR 12 (veterans RAND 12)

The VR-12 is a Patient Reported Outcome instrument that uses 12 questions to capture the global health status of patients across 8 health domains. This is possible regardless of the patient's disease.

For the evaluation of the VR-12, one physical and one mental total score are calculated: “Physical Component Summary (PCS)” and “Mental Component Summary (MCS)”.

It is a validated and established score, which is very well suited for implementation in clinical research and patient care due to its short length, strong validity and universal applicability (Kwon and Sawatzky, 2017; Johnson and Maddigan, 2004; Feeny et al., 2005; Vallance et al., 2013).

### 2.6 Data analysis

SPSS® (IBM Corp. Released 2020. IBM SPSS Statistics for Macintosh, Version 27.0. Armonk, NY: IBM Corp) was used to conduct the analysis. One-way analysis of variance (ANOVA) with repeated measurements was utilized to detect mean differences in the independent variables of Time (baseline, 1, 3, 6 and 12 months) for the variables of VAS and KOOS.

Descriptive statistics where used to describe the patient base and the number of nucleated cells injected. For the correlation of the number of injected cells and clinical outcome 3 Patients with a cell count above 400 million cells were excluded from the analysis as extreme outliers.

The unpaired *t*-test was applied to compare number und viability as well as the improvement of the clinical outcome between male and female patients. To quantify the clinical improvement in each outcome score, baseline values were subtracted from the score at 6 months for each patient.

The clinical improvement after 6 months was correlated with the number of injected cells and their viability using linear regression and person correlation.

### 2.7 Descriptive statistics

18 male and 15 female patients aged 23 to 88 years with a mean age of 60.58 years were included. 3 male patients received bilateral SVF injection.

In a total of 25 patients the number of injected cells and in 21patients the percentage of vital cells could be determined. In the remaining 4 patients the number of vital cells could not be determined due to necessary adjustments of the measurement system.

## 3 Results

The number of nucleated cells injected ranged between 6.61 million and 98.5 million cells with a mean of 44.9 million cell. The average percentage of vital cells was 64.43% with a range of 38.7% to 89.9%.

All patients suffered from gonarthrosis with a KL score of 1–4. All patients were treated under the same conditions by the same orthopedic surgeon.

### 3.1 VAS

**Figure:**
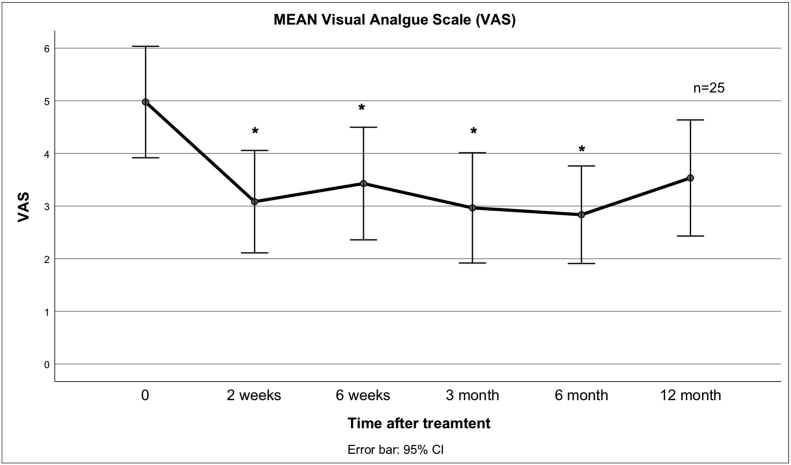
Fig. 2 describes the mean VAS score at different times after treatment^1^ there are Significant changes up to the 6 months measurement time point. ^1^The * indicates a significant difference compared to when compared to the baseline values.

### 3.2 KOOS

**Figure:**
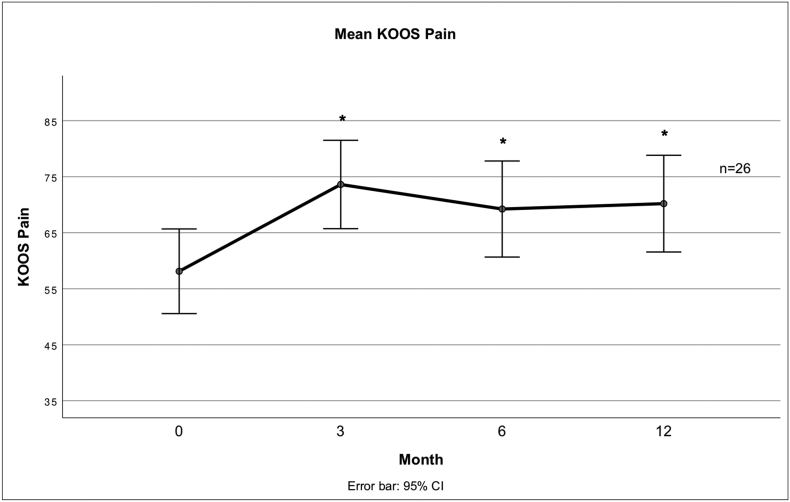
Fig. 3 describes the mean score for the Pain subscale of the KOOS at different times after treatment.

**Figure:**
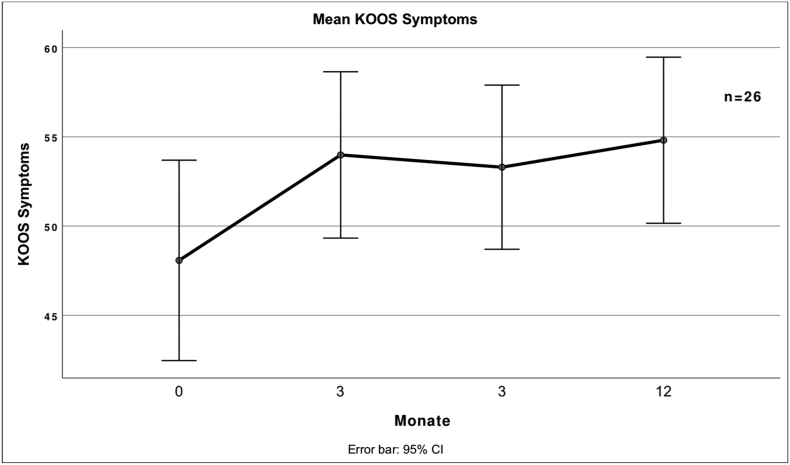
Fig. 4 describes the mean score for the symptom's subscale of the KOOS at different times after treatment.

**Figure:**
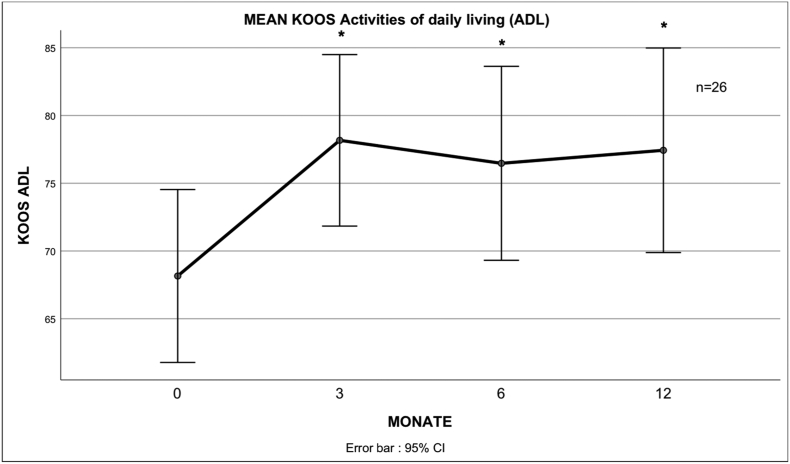
Fig. 5 describes the mean score for the ADL subscale of the KOOS at different times after treatment.

**Figure:**
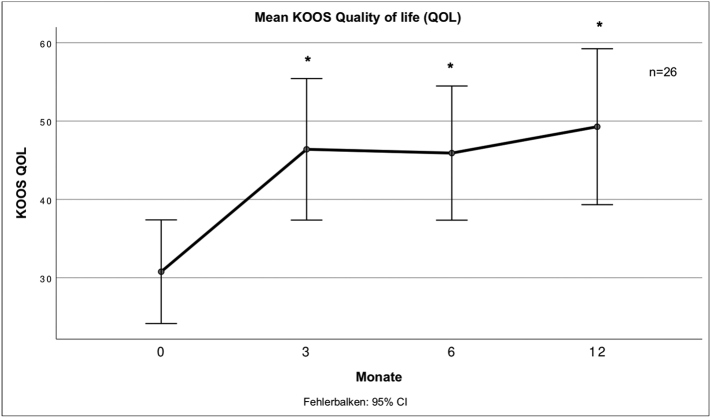
Fig. 6 describes the mean score for the QOL subscale of the KOOS at different times after treatment.

**Figure:**
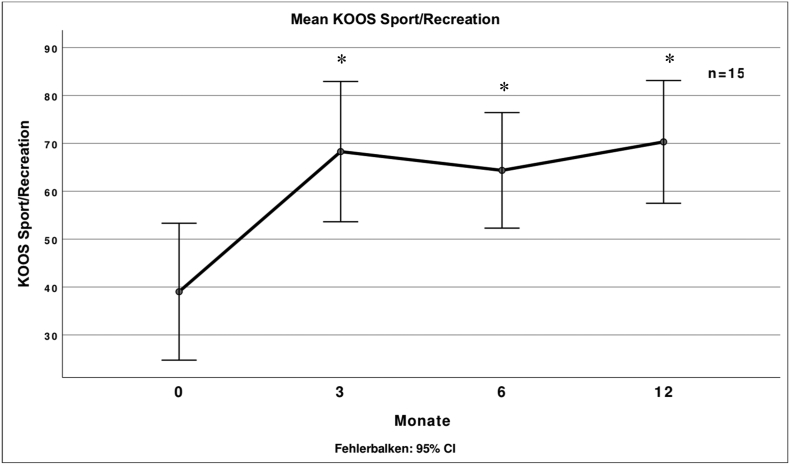
Fig. 7 describes the mean score for the Sport/Rec subscale of the KOOS at different times after treatment.

All subscales of the KOOS showed significant improvement to the baseline values except the symptom's subscale. There was no significant further improvement after 3 months.

### 3.3 VR 12

**Figure:**
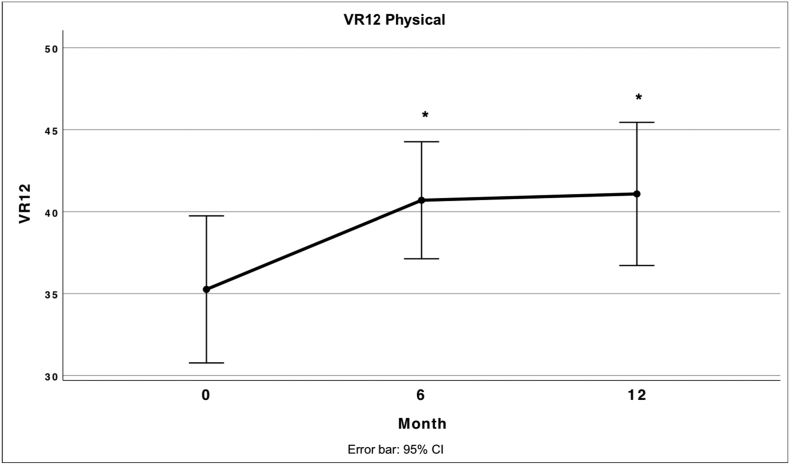
Fig. 8 Describes the mean score for the Physical Component Summary of the VR12 at different times after treatment.

**Figure:**
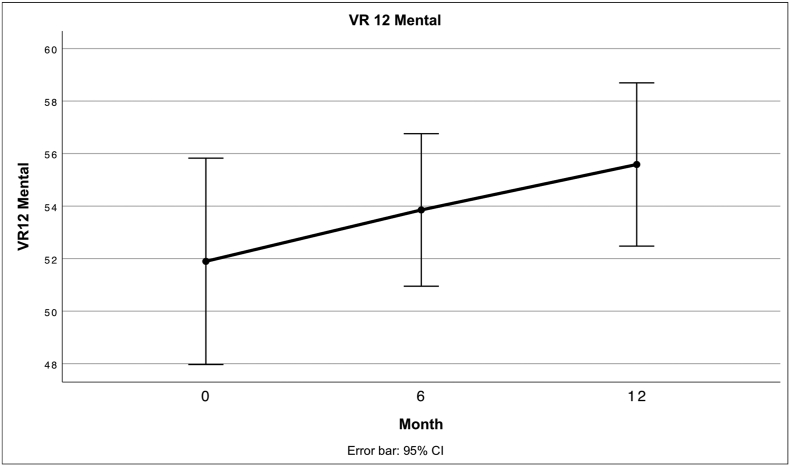
Fig. 9 Describes the mean score for the Mental Component Summary of the VR12 at different times after treatment.

### 3.4 Comparison of number of injected cells, viability and treatment outcome of male and female patients

**Figure:**
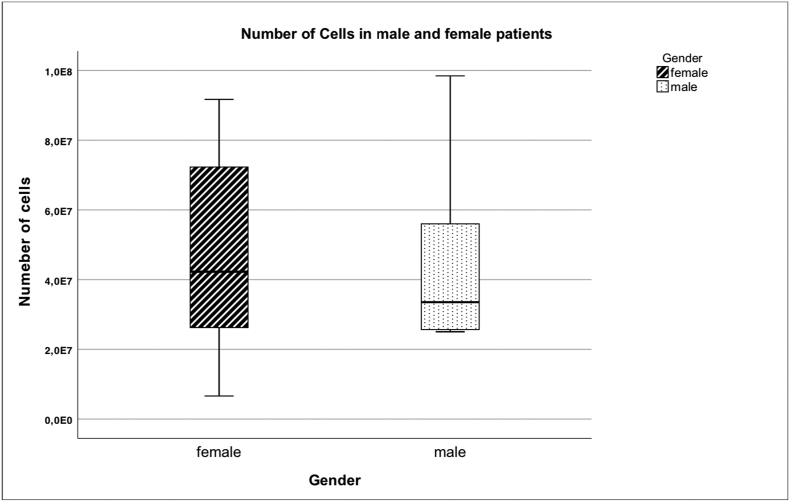
Fig. 10 Mean number of cells in male and female patients there is no significant difference between male and female patients.

**Figure:**
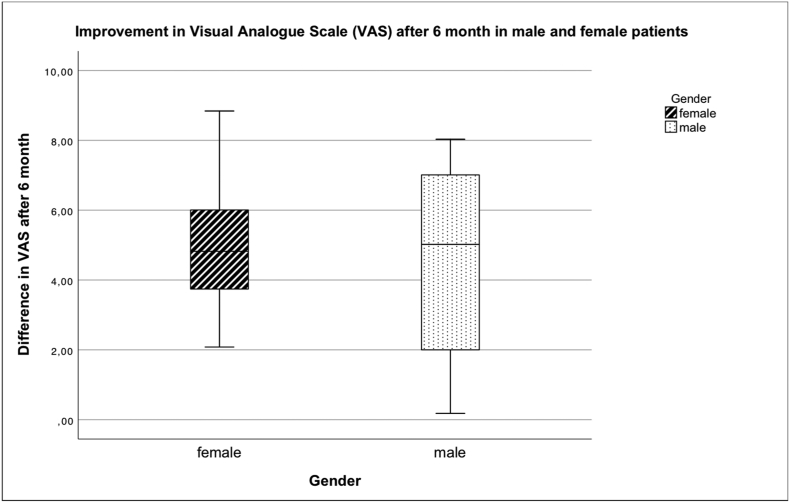
Fig. 11 Improvement of the VAS score after 6 months. There was no statistically significant difference between male and female patients.

There was no statistically significant difference between male and female patients in cell count and vitality as well as in the improvement of KOOS, VAS and VR-12.

### 3.5 Correlation of cell number and viability with treatment success

**Figure:**
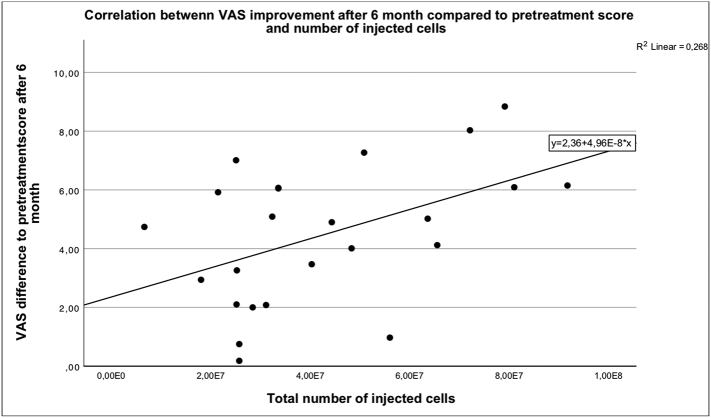
Fig. 12 shows a statistically significant correlation with the number of injected cells and improvement in VAS after 6 Month.

There was no significant correlation between the number of injected cells and the individual subscales of the KOOS.

**Figure:**
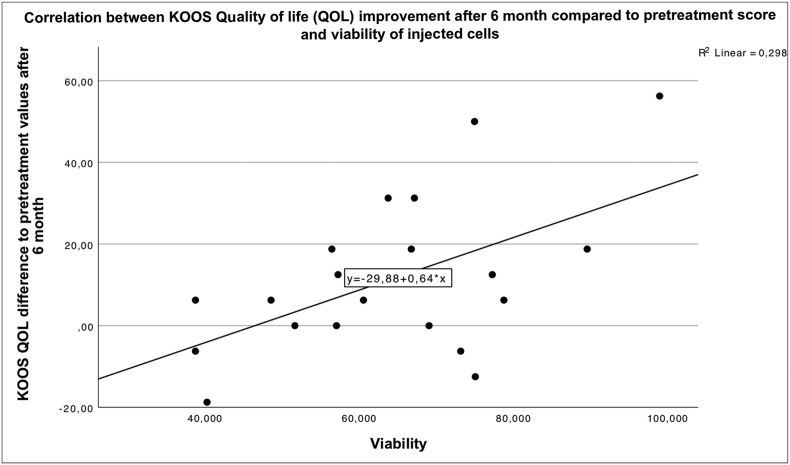
Fig. 13 shows a significant correlation between the improvement of the QOL subscale of the KOOS and the vitality of the injected cells.

There was no statistically significant correlation between the viability of cells and the individual VAS as well as the remaining four subscales of the KOOS or the SF-12.

### 3.6 Complications

No major complications occurred in any of the patients.

## 4 Discussion

### 4.1 Interpretation of results and comparison to other studies

Due to the high lifetime prevalence in combination with high suffering pressure, the therapy of gonarthrosis plays a decisive role in clinical routine. In addition to conservative therapy options, many patients, especially in advanced stages, can only be offered the implantation of a knee joint prosthesis with corresponding peri- and postoperative risks.

In recent years, therefore, cell therapy, by which cartilage regeneration can be induced, has come to play an increasingly important role (Freitag et al., 2019; Tsubosaka et al., 2020; Fodor and Paulseth, 2015; Giannoudis et al., 2016). However, since this is strictly regulated due to European legislation, few prospective data are available. The approach we have chosen provides further prospective data using a technique that is in line with existing European regulations. We present a prospective case study investigating the clinical outcome after injection of SVF into the Hoffa's fat pad as well as the cell count and viability of SVF produced by mechanical processing of autologous fat tissue. Previous studies on the use of SVF for the therapy of gonarthrosis are based on an enzymatic process for the production of SVF as well as its intra-articular injection. In contrast, our method is based on a mechanical manufacturing process and the choice of IFP as the target tissue for which only an authorization for homologous use of SVF is required in Germany. The purpose of this pilot study was to evaluate the efficacy and therapeutic benefit of this new therapeutic procedure. The number of included patients is with 33 comparatively large and sufficient for the establishment of the method. We used well-established and internationally known outcome scores to monitor the progress of therapy. Because of the invisivity and possible risks associated with fat sampling, we did not include a placebo group for ethical reasons.

Both the VAS and the respective subscales of the KOOS except the symptom's subscale show a significant improvement compared to the baseline values. Especially in the subscales of the KOOS, which represent function and quality of life, a significant improvement was achieved. In this regard, our results are comparable to other studies in which enzymatically produced SVF or culture expanded ADSCs were injected into the knee (Freitag et al., 2019; Tsubosaka et al., 2020; Fodor and Paulseth, 2015). PCS-12 also showed significant improvements compared to baseline, while the MCS-12 showed no significant change which is in line with expectations.

There was no gender-specific difference in the number of injected cells, their viability or the success of the therapy.

The number of injected cells showed a high variance This is primarily due to the different cell concentrations in one milliliter of injection solution and, of course, to the total amount of starting material removed. In a recently published study from Japan, patients were injected with an SVF preparation containing almost twice the number of cells compared to our preparation (mean of 76 million cells). In Japan, the Celution® 800/CRS system (Cytori Therapeutics Inc., San Diego, CA) was used. With 334.3 ± 44.0 ml, a much larger amount of lipoaspirate was collected to produce SVF. For this comparatively more complex procedure, the patients received general anesthesia (Tsubosaka et al., 2020). In the system we used, a maximum of 30 ml of lipoaspirate was collected under tumescent anesthesia. As expected, treatment with SVF does not yield preparations with a constant cell count. Besides technical and systematic differences, patient-related variables may also be decisive. In order to maximise the effect on the patients, the complete amount of SVF was injected without adjusting the amount of Cell to specific concentration after the cell count. The therapeutic benefit of different cell doses will be subject of future follow-up studies with increased initial collection volumes. As expected, the VAS score showed a significant correlation with the number of injected cells, whereas the change in KOOS could not be correlated. In this very heterogeneous patient population with respect to age and degree of arthritis, it can be assumed that besides the number and viability of the cells, there are other factors that can influence the response to therapy. However, since a significant correlation was found, it can be assumed that the number of injected cells is a significant influencing factor.

In several dose escalation studies, a clear relationship was found between higher cell doses and improved clinical outcome and cartilage regeneration (Jo et al., 2014; Fodor and Paulseth, 2015; Song et al., 2018).

Freitag J. et al. presented a 2019 study in which in vitro cultured ADSCs were injected. In this study, 100E+06 ADSCs were administered per injection. The patients were divided into three randomized groups. One group received only one injection, the second group received an additional injection after 6 months and the third group received a placebo. The results at 6 months of the first two groups in pain and function were comparable to ours and significantly better than in the placebo group. There were no significant differences in pain and function between the single and double injection groups, even after one year. In the two-injection group, however, radiological evidence of an improvement in cartilage quality could be found after one year, which was interpreted as an argument for greater therapeutic efficiency when injected again after 6 months. However, as the cell products used in the already published studies differ greatly in terms of cell processing, the values determined cannot be used for a general dose recommendation (Freitag et al., 2019).

The viability of the injected cells showed a very wide range and comparatively low viability. However, this measurement is biased by the fact that the time between cell collection and cell measurement varied between one and 5 h during which the preparation was stored at room temperature. Nevertheless, a correlation between a high percentage of vital cells and an improvement of the QOL subscale of the KOS could be measured. It can be assumed that the highest possible percentage of vital cells should be aimed for.

### 4.2 Conclusion

Our procedure represents a safe and minimally invasive option for the treatment of gonarthrosis with a KL score up to 4 at initial results. Significant relief of pain as well as significant improvement in function were achieved. Compared to other procedures, in which patients received general anesthesia or the cells first had to be expanded in vitro, the effort and the associated psychological and physical stress for the patients could be reduced to a minimum.

Although there is no reliable information regarding the optimal cell dose in the current limited study situation, our results suggest that the number of injected cells is within a therapeutically effective range.

### 4.3 Limitations and outlook

At this point in time, most of the available information consists of studies with very low case numbers. There is no uniform standard with regard to cell processing. This pilot study was not randomized and did not have a control group and was designed primarily to establish the method and to test its safety.

Within the next steps, a randomized study with a control group should be performed. To avoid an ethical dilemma, a design could be chosen in which patients with bilateral gonarthrosis could serve as their own control group. One knee could be injected with SVF and the other with a saline solution. However, this would require finding patients with a comparable degree of osteoarthritis in both knees. Although the strong heterogeneity of our patient population reflects the clinical routine, it represents a rigid limitation, especially with regard to dose determination. The elimination of unintended influences by a more homogeneous patient population will be essential in future dose-finding studies. In order to obtain a more reliable statement about the viability of the cells, vitality tests would have to be carried out immediately after the injection of the cells. A comparative study with an already established method of gonarthrosis therapy such as intra-articular injection of hyalonic acid would be helpful in establishing individual treatment strategies. In the future, osteoarthritis treatment with cell products such as SVF could not only play a decisive role in delaying the need for joint replacement. It would also be possible to use the method after injuries that have an increased risk of osteoarthritis development such as meniscus or cruciate ligament lesions.

## CRediT authorship contribution statement

**Klaus Werner Labarre:** Conceptualization, Methodology, Formal analysis, Investigation, Data curation, Writing – original draft, Writing – review & editing, Visualization, Supervision, Project administration. **Gerald Zimmermann:** Conceptualization, Methodology, Validation, Resources, Writing – review & editing, Supervision, Project administration, Funding acquisition.

## Declaration of competing interest

There is no conflict of interest for Klaus Werner Labarre. Prof. Dr. med. Gerald Zimmermann has a consulting agreement with the company Arthrex. There was no funding with regard to this study.

## Footnotes

1 This is a cannula from *Tulip Medical Products* (*San Diego*, *CA*, *USA*) with a blunt end and 3 lateral openings which has been developed for cell-sparing multi-planar harvesting of adipose tissue.
